# P03.13. Assessment of student learning at a CAM institution - why and how?

**DOI:** 10.1186/1472-6882-12-S1-P266

**Published:** 2012-06-12

**Authors:** S Vinjamury, G Thompson, M Nagare, K Rose

**Affiliations:** 1 Southern California University of Health Sciences, Whittier, USA

## Purpose

The purpose of this paper is to describe the importance of proper assessment of student learning and the process adopted by a CAM institution in the establishment of Student Learning Outcome Departments to assess student learning.

## Methods

Student Learning Outcomes (SLOs) are specific statements that describe the knowledge, skills and attitudes that students are expected to learn at the successful completion of the curriculum. They provide the students with what to expect of the program and themselves. SLOs also assist in programmatic review, evaluation and improvement of the curriculum. As a result of their extensive role, outcome based education has become a priority in health professions. While it has been successfully implemented in medical, dental and nursing schools, outcome based education is gradually evolving in the complementary and alternative medicine professions. Our institution created four exclusive SLO Departments to develop, refine, and manage SLOs across all the programs. Furthermore, these departments were given the responsibility of assessment of student learning.

## Results

The development of SLOs occurred in two phases: (1) Faculty training that focused on the significance of learning based curriculum and the first draft of SLOs were created by faculty at large. (2) Establishment of four exclusive departments that are headed by Department Chairs. These departments were populated with both pre-clinical and clinical faculty across both programs at our institution. These assignments were in addition to their primary academic affiliation to a department. The Department Chairs and the faculty work together to ensure that SLOs are being assessed and that Student performance on the SLOs is tracked.

## Conclusion

SLOs provide a framework by which an institution’s efforts, goals, mission, and objectives are made explicable to both internal and external constituencies. Independent SLO Departments may be helpful in assessing student learning better as it is their exclusive task.

